# Relationship Between Illness Representations and Symptoms of Internet Gaming Disorder Among Young People: Cross-Lagged Model

**DOI:** 10.2196/28117

**Published:** 2021-11-30

**Authors:** Xue Yang, Kei Man Wong, Rui She, Chengjia Zhao, Nani Ding, Huihui Xu, Xiaolian Tu, Xinyi Lai, Guohua Zhang

**Affiliations:** 1 JC School of Public Health and Primary Care Faculty of Medicine The Chinese University of Hong Kong Hong Kong China (Hong Kong); 2 Department of Psychology School of Mental Health Wenzhou Medical University Wenzhou China; 3 School of Public Health and Management Wenzhou Medical University Wenzhou China; 4 Renji College Wenzhou Medical University Wenzhou China; 5 The Affiliated Kangning Hospital Wenzhou Medical University Wenzhou China; 6 Institute of Aging Key Laboratory of Alzheimer's Disease of Zhejiang Province Wenzhou Medical University Wenzhou China

**Keywords:** illness representations, internet gaming disorder, youth, cross-lagged model

## Abstract

**Background:**

The common-sense model of illness suggests that mental representations of health threats may affect one’s behavioral reactions to them and health status. Internet gaming disorder is a newly defined mental disorder. Illness representations of internet gaming disorder may affect one’s risk of internet gaming disorder. In turn, symptoms of internet gaming disorder may affect one’s perceptions of the disorder.

**Objective:**

This study aimed to investigate the relationships between illness representations and symptoms of internet gaming disorder in college students.

**Methods:**

A 1-year longitudinal study was conducted with a convenience sample of Chinese college students (n=591; 342/591, 57.9% female).

**Results:**

Of the participants, 10.1% (60/591) and 9.1% (54/591) were classified as having probable internet gaming disorder at baseline (T1) and follow-up (T2), respectively. The correlations between some dimensions of illness representations regarding internet gaming disorder (ie, consequence, timeline, personal control, treatment control, and concern) at T1 and symptoms of internet gaming disorder at T2 and between symptoms of internet gaming disorder at T1 and the dimensions of illness representations at T2 (ie, consequence, timeline, personal control, and emotional response) were statistically significant. The cross-lagged model fit the data well ((χ^2^/*df*=2.28, comparative fit index=.95, root mean square error of approximation=.06) and showed that internet gaming disorder at T1 was positively associated with unfavorable illness representations at T2.

**Conclusions:**

Individuals with more severe symptoms of internet gaming disorder had more pessimistic perceptions about the disorder. Such cognitive perceptions may affect one’s emotional and behavioral reactions towards the disorder (eg, greater levels of depression and low self-control intention) and should be modified by educational programs and psychological interventions.

## Introduction

### Background

Internet gaming is the most common leisure activity among young people in East Asian countries, including China [1-4]. Although some studies have proposed positive effects of internet gaming [5,6], excessive internet game use frequently leads to various physical (eg, obesity), psychological (eg, depression, anxiety), and social problems (eg, interpersonal conflict) [1-3]. Internet gaming disorder (IGD) is defined as “persistent and recurrent use of the internet to engage in games, often with other players, leading to clinically significant impairment or distress” [7,8]. It was listed as a condition for further research in the Diagnostic and Statistical Manual of Mental Disorder (DSM-5) [7] and later as an official disorder in the 11th Revision of the International Classification of Diseases (ICD-11) [9]. Examples of IGD symptoms include preoccupation, tolerance, and inability to control gaming behaviors in the past year [7,9]. Debates have continued about the conceptualization, diagnostic criteria, and guidelines for IGD [8].

Prevalence rates of IGD vary widely across studies, ranging from 0.7% to 15.6% [10]. A recent meta-analysis found a worldwide IGD prevalence of 3.05% [11]. Youth and young adults are high-risk populations for IGD. For example, a cross-cultural study among young adults (aged 18-25 years) reported IGD prevalence rates of 26% in the United States, 21.1% in China, and 15.4% in Singapore [12]. Studies among Chinese college students found prevalences ranging from 4% to 25.7% [13-17]. A longitudinal survey with Chinese university students reported prevalence rates of 14.8% and 9.9% at baseline and the 1-year follow-up, respectively [18]. IGD has been found to be associated with other mental disorders, such as depression, anxiety, and attention-deficit/hyperactivity disorder [19-22] as well as health and life problems, including poor sleep quality, academic failure, and interpersonal problems [16,23,24]. Given the high prevalence and severe consequences of IGD in young people, efforts to understand how they perceive this newly defined disorder is warranted. The understanding of these perceptions and relationships would facilitate related research and intervention development (eg, educational programs, psychological interventions) to prevent and reduce IGD.

### Concepts of Illness Representations

The lay understanding of a potential health threat (ie, illness representations) may influence individuals’ responses to it (eg, IGD). The common-sense model (CSM) of illness [25] suggests that when individuals encounter a health threat, they develop mental representations of it based on their general knowledge and past experience, information from the external social environment (eg, friends), and current experience of it. Illness representations involve cognitive and emotional representations. To be specific, the cognitive representations include consequences (assessing the severity of the illness), timeline (considering whether the illness is acute/chronic/cyclical), control (considering whether the illness is under volitional control), identity (labeling the illness and identifying its symptoms), illness comprehension (overall comprehension of the illness), and cause (attributing likely causes of the illness). Emotional representations (expressing the emotions evoked by the illness) typically entail negative emotional responses such as anger and worry. These representations interact with each other and work in parallel to guide individuals’ coping strategies and affect their mental and behavioral responses to the illness (eg, self-management, help-seeking). For example, it was found that high treatment control (ie, perceived treatment effectiveness) was associated with better treatment adherence among patients with bipolar disorder [26] and type 2 diabetes [27]. Illness representations are dynamic. They would be appraised and revised based on the consequences of the coping responses to health threats [25] and could be modified by interventions [28,29].

### Relationships Between Illness Representations and Symptoms of IGD

Illness representations of IGD may affect one’s risk of IGD. People with unfavorable illness representations of IGD (eg, perceiving and worrying about severe consequences of IGD) may be less likely to have IGD. It may be because such people are more likely to regulate their internet gaming behaviors, prevent themselves from excessive internet gaming and IGD, or make an effort to reduce their IGD symptoms. Although related studies are limited, CSM can be used to support our hypothesis theoretically, which postulates that both cognitive representations and emotional representations of the illness can affect coping and appraisal of a disease, which in turn determine health-related outcomes [25,30]. In addition, a number of studies have investigated the associations between illness representations and various health-related behaviors (eg, self-management behaviors, willingness to consult health care providers) and health outcomes (eg, physical functioning, mental health status, and clinical health outcomes), among both patients and healthy populations [30-32]. For example, a previous study reported that illness representations of H1N1 were significantly associated with H1N1 preventive behaviors, including wearing face masks, hand washing, and behavioral intention to take up influenza vaccination [32].

However, IGD symptoms may increase the levels of unfavorable representations of IGD. It may be because people with greater symptoms of IGD may have experienced negative consequences of IGD or had trouble with regulating their internet gaming behaviors, thus enhancing their unfavorable representations of IGD, while those with no or minimal symptoms of IGD have not experienced negative consequences of IGD or may not necessarily think about IGD negatively [33]. A recent cross-sectional study in an adult sample (mean age 40.42 years) in Macao, China reported that those with IGD showed more negative emotional representations, scored higher in the level of the time cyclical factor, and had better comprehensiveness of IGD than non-IGD cases [33]. However, this was the only existing study, and the cross-sectional study design could not demonstrate a causal relationship between IGD and illness representations of IGD. To our best knowledge, no study has investigated the longitudinal and interactive relationships between illness representations and symptoms of IGD. This study attempted to fill this gap.

### This Study

This 2-wave longitudinal study aimed to understand how young people cognitively and emotionally perceive IGD as a disorder and health threat (ie, illness representations). In addition, the study investigated the relationships between such perceptions and symptoms of IGD in a population of Chinese college students. It was hypothesized that (1) unfavorable illness representations (eg, severe consequence) of IGD at baseline (T1) would predict low levels of IGD symptoms at follow-up (T2); however, (2) low levels of IGD symptoms at baseline (T1) would predict high levels of unfavorable illness representations of IGD at follow-up (T2).

## Methods

### Participants

This longitudinal study adopted a convenience sample recruited from 2 universities in China. The inclusion criteria of this study included (1) being a first-year college student, (2) being willing to participate in the baseline and follow-up studies, and (3) playing internet games in the past year. The exclusion criterion was being a non-Chinese speaker. Data from the participants who completed the 2-wave surveys were reported in this study (n=591). As shown in Table 1, about three-fifths of the participants (342/591, 57.9%) were female, 51.4% (287/591) were from urban areas, and 48.7% (288/591) lived in one-child families. The mean hours of internet gaming per week were 7.7 (SD 9.7) at T1 and 8.4 (SD 10.7) at T2.

**Table 1:** Background characteristics of the participants (n=591).

Background characteristics			Participants, n (%)
**Sex**			
	Female	342 (57.9)	
	Male	249 (42.1)	
**Residence**			
	Urban	304 (51.4)	
	Rural	287 (48.6)	
**One-child family**			
	Yes	288 (48.7)	
	No	303 (51.3)	
**Hours of internet gaming per** **week**			
	0-7	264 (44.6)	
	>7 to 14	181 (30.7)	
	>14 to 21	85 (14.3)	
	>21	67 (11.4)	
**Major**			
	Anesthesia	84 (14.2)	
	Forensic Medicine	87 (14.7)	
	Stomatology	165 (27.9)	
	Chinese Medicine	137 (23.1)	
	Psychology	118 (20.0)	

### Data Collection

The baseline survey (T1) was conducted during the first year of college study of the participants, while the 1-year follow-up survey (T2) was conducted during their second year of college study. In the absence of any teacher, the surveys were conducted in classroom settings. A research assistant with 2 years of experience in data collection and who majored in psychology assisted with data collection and answered any questions during the survey. The participants were assured that participation was voluntary and refusals would have no negative consequences. Data confidentiality was guaranteed, and only the researchers could access the data. Student IDs were used for data matching. Researchers were not able to access students’ names or other identifying information. All participants were briefed on the purpose of the study and provided their informed consent to participate in this anonymous survey. They were also provided with information of local psychological services in case it was needed. The study procedures were carried out in accordance with the Declaration of Helsinki. Ethical approval was obtained from the affiliated university of the corresponding author.

### Measures

#### Illness Representations of IGD

The Brief Illness Perception Questionnaire (B-IPQ) was used to assess the participant's cognitive and emotional perceptions of IGD (Multimedia Appendix 1). The B-IPQ has 8 dimensions; each is measured by 1 item [34]. The items are related to consequences, timeline, personal control, treatment control, identity, illness concern, comprehension, and emotional response. A sample item is “How much does IGD affect the life of an IGD gamer?”: 0 (no effect at all) to 10 (severely affects my life). The items have demonstrated adequate test-retest reliability and predictive validity [35]. The Chinese version of the IPQ regarding IGD (IPQ-IGD) has been validated in Chinese adults [33]. Confirmatory factor analysis showed acceptable model fit of B-IPQ at T1 (χ^2^:df=2.50, comparative fit index [CFI]=.92, root mean square error of approximation [RMSEA]=.07, and standardized root mean square residual [SRMR]=.06) and at T2 (χ^2^:df=2.91, CFI=.94, RMSEA=.06, SRMR=.05). Composite reliability was .79 at T1 and .80 at T2. Average variance extracted was .30 at T1 and .31 at T2. Cronbach α was.71 at T1 and .73 at T2.

#### IGD Symptoms

IGD symptoms were assessed using the 9 diagnostic criteria proposed in the DSM-5 (Multimedia Appendix 1) [7]. It is a short, user-friendly, self-report measure assessing IGD symptoms of preoccupation, tolerance, withdrawal, unsuccessful attempts to limit gaming, deception or lies about gaming, loss of interest in other activities, use despite knowledge of harm, use for escape or relief of negative mood, and harm based on DSM-5 criteria [7]. Participants answered whether they had experienced symptoms in the last 12 months (0=No, 1*=*Yes). Higher total scores indicate severer IGD symptoms. Participants who scored ≥5 were classified as having probable IGD [36]. The internal reliability was 0.86 at T1 and 0.80 at T2. The results showed that the intraclass correlation coefficient was 0.70, suggesting moderate test-retest reliability. The tool has been widely used in other IGD studies [1-3]. Like previous studies [2], the continuous variable of IGD was used in the data analyses [1,36,37]. The Chinese version of the scale was found to have good psychometric properties and is widely used in Chinese populations [1,36,37].

#### Statistical Analysis

Descriptive statistics of and Pearson’s correlation analyses between the continuous variables of each dimension of B-IPQ and IGD were performed using SPSS 25.0 (IBM Corp, Armonk, NY). The level of statistical significance was set at .05. Furthermore, the cross-lagged model regarding the relationships between all the dimensions of B-IPQ and the IGD score was performed by structural equation modeling using SPSS Amos 25. Latent variables of B-IPQ were created with the scores of each dimension of B-IPQ being used as observed variables. The variables at the same time point were covariated. The goodness-of-model fit was assessed using the Chi-square:degrees of freedom (χ^2^:df) ratio, CFI, RMSEA, and SRMR. A χ^2^:df ratio ≤3, CFI ≥.90, RMSEA ≤.08, and/or SRMR ≤.08 would indicate acceptable model fit [38,39].

## Results

### Descriptions of B-IPQ at T1 and T2

As shown in Table 2, at T1, one-fifth of the participants perceived that IGD would severely affect his or her life (121/591, 20.4%) or would last forever (120/591, 20.3%); 45.9% (271/591) of the participants indicated that IGD would lead to severe symptoms. More than half (343/591, 58.1%) of the participants would be concerned about IGD. More than half (338/591, 57.2%) perceived personal control over IGD, while less than half of the participants believed that treatment of IGD could control the disease (241/591, 40.8%) and felt that they understood IGD (268/591, 45.3%). Only 37.2% (220/591) of the participants indicated that they would develop negative emotions due to IGD.

However, the percentages of item agreement increased at T2. To be specific, 43.0% (254/591) of the participants perceived that IGD would severely affect his or her life; 25.5% (151/591) believed that IGD would last forever; 60.3% (356/591) of the participants indicated that IGD would lead to severe symptoms; and 74.0% (437/591) would be concerned about IGD. Although 68.7% (406/591) perceived personal control over IGD, more than half of the participants believed that treatment of IGD could control the disease (333/591, 56.3%) and felt that they understood IGD (308/591, 52.1%). More than half (308/591, 52.1%) indicated that they would develop negative emotions due to IGD.

**Table 2:** Number of participants who endorsed each item of the Brief Illness Perception Questionnaire (B-IPQ; score ≥6) at T1 (baseline) and T2 (1-year follow-up; n=591).

B-IPQ items	T1, n (%)	T2, n (%)
Item 1: consequences	121 (20.4)	254 (43.0)
Item 2: timeline	120 (20.3)	151 (25.5)
Item 3: personal control	338 (57.2)	406 (68.7)
Item 4: treatment control	241 (40.8)	333 (56.3)
Item 5: identity	271 (45.9)	356 (60.3)
Item 6: concern	343 (58.1)	437 (74.0)
Item 7: comprehension	268 (45.3)	308 (52.1)
Item 8: emotional response	220 (37.2)	308 (52.1)

### Descriptions of IGD at T1 and T2

Of the participants, 10.1% (60/591) and 9.1% (54/591; *P*=.64) of the participants were classified as having probable IGD at T1 and T2, respectively. Among those without IGD at T1, 4.3% (23/531) were classified with IGD (incidence rate), while among those with IGD at T1, 53.8% (32/60) remitted from IGD at T2 (remission rate).

### Correlations Between B-IPQ and IGD Symptoms

As shown in Table 3, the dimensions of consequence (*r*=0.16), timeline (*r*=0.27), personal control (*r*=–0.15), and emotional response (*r*=0.10) of IPQ at T1 were significantly correlated with IGD at T2 (*P*<.05). IGD at T1 was significantly correlated with the dimensions of consequences (*r*=0.17), timeline (*r*=0.38), personal control (*r*=–0.30), treatment control (*r*=–0.10), and concern (*r*=–0.12) of IPQ at T2.

**Table 3:** Correlations between Brief Illness Perception Questionnaire (B-IPQ) dimensions and internet gaming disorder (IGD) symptoms at T1 (baseline) and T2 (1-year follow-up).

Variable	Consequence-T1	Timeline-T1	Personal control-T1	Treatment control-T1	Identity-T1	Concern-T1	Comprehension-T1	Emotional response-T1	Consequence-T2	Timeline-T2	Personal control-T2	Treatment control -T2	Identity-T2	Concern-T2	Comprehension-T2	Emotional response-T2	IGD symptoms-T1	IGD symptoms-T2
**Consequence-** **T1**																		
*r*	1	0.61	0.08	0.19	0.47	0.25	0.13	0.40	0.19	0.13	0.00	–0.04	0.12	0.13	0.15	0.18	0.23	0.16
*P* value	—^a^	<.001	.33	.02	<.001	<.001	.12	<.001	.03	.13	.99	.63	.17	.13	.09	.04	.01	.07
**Timeline-T1**																		
*r*	0.61	1	–0.11	0.06	0.18	–0.03	0.03	0.19	0.22	0.30	–0.13	–0.05	0.00	–0.08	–0.06	0.07	0.29	0.27
*P* value	<.001	—	.20	.51	.03	.75	.69	.02	.01	<.001	.15	.56	.98	.37	.53	.45	<.001	<.001
**Personal control-T1**																		
*r*	0.08	–0.11	1	0.49	0.38	0.36	0.36	0.29	–0.07	–0.25	0.17	–0.03	–0.09	–0.11	0.08	–0.10	–0.17	–0.15
*P* value	.33	.20	—	<.001	<.001	<.001	<.001	<.001	.40	<.001	.06	.76	.31	.23	.36	.23	.02	.08
**Treatment control-T1**																		
*r*	0.19	0.06	0.49	1	0.54	0.52	0.31	0.47	0.07	–0.03	–0.01	0.27	0.03	0.11	0.02	–0.04	–0.03	–0.02
*P* value	.02	.51	<.001	—	<.001	<.001	<.001	<.001	.42	.73	.92	<.001	.77	.20	.80	.67	.75	.79
**Identity-T1**																		
*r*	0.47	0.18	0.38	0.54	1	0.58	0.39	0.48	0.29	0.07	–0.01	–0.05	0.08	0.11	0.15	0.15	0.06	0.05
*P* value	<.001	.03	.00	<.001	—	<.001	<.001	<.001	<.001	.44	.88	.57	.37	.22	.10	.08	.36	.57
**Concern-T1**																		
*r*	0.25	–0.03	0.36	0.52	0.58	1	0.51	0.53	0.33	0.00	0.09	0.16	0.10	0.23	0.21	0.27	–0.09	–0.03
*P* value	<.001	.75	<.001	<.001	<.001	—	<.001	<.001	<.001	.96	.31	.07	.28	.01	.02	<.001	.29	.77
**Comprehension-T1**																		
*r*	0.13	0.03	0.36	0.31	0.39	0.51	1	0.39	0.21	0.05	0.12	0.09	0.00	0.16	0.27	0.21	–0.14	–0.02
*P* value	.12	.69	<.001	<.001	<.001	<.001	—	<.001	.02	.60	.17	.34	.99	.07	<.001	.02	.10	.86
**Emotional response-T1**																		
*r*	0.40	0.19	0.29	0.47	0.48	0.53	0.39	1	0.27	0.18	0.06	0.17	0.13	0.13	0.15	0.17	0.06	0.10
*P* value	<.001	.02	<.001	<.001	<.001	<.001	<.001	—	<.001	.05	.13	.06	.14	.16	.10	.05	.48	.24
**Consequence-** **T2**																		
*r*	0.19	0.22	–0.07	0.07	0.29	0.33	0.21	0.27	1	0.62	0.05	0.28	0.49	0.43	0.39	0.53	0.17	0.25
*P* value	.03	.01	.40	.42	<.001	<.001	.02	<.001	—	<.001	.53	<.001	<.001	<.001	<.001	<.001	.05	<.001
**Timeline-T2**																		
*r*	0.13	0.30	–0.25	–0.03	0.07	0.00	0.05	0.18	0.62	1	–0.12	0.20	0.42	0.22	0.13	0.40	0.38	0.45
*P* value	.13	<.001	<.001	.73	.44	.96	.60	.05	<.001	—	.17	.02	<.001	.01	.12	<.001	<.001	<.001
**Personal control-T2**																		
*r*	0.00	–0.13	0.17	–0.01	–0.01	0.09	0.12	0.06	0.05	–0.12	1	0.51	0.23	0.41	0.32	0.22	–0.30	–0.19
*P* value	.99	.15	.06	.92	.88	.31	.17	.13	.53	.17	—	<.001	.01	<.001	<.001	.01	<.001	.05
**Treatment control -T2**																		
*r*	–0.04	–0.05	–0.03	0.27	–0.05	0.16	0.09	0.17	0.28	0.20	0.51	1	0.33	0.49	0.22	0.32	–0.10	–0.01
*P* value	.63	.56	.76	<.001	.57	.07	.34	.06	<.001	.02	<.001	—	<.001	<.001	.01	<.001	.24	.90
**Identity-T2**																		
*r*	0.12	0.00	–0.09	0.03	0.08	0.10	0.00	0.13	0.49	0.42	0.23	0.33	1	0.52	0.32	0.59	0.06	0.16
*P* value	.17	.98	.31	.77	.37	.28	.99	.14	<.001	<.001	.01	<.001	—	<.001	<.001	<.001	.06	.06
**Concern-T2**																		
*r*	0.13	–0.08	–0.11	0.11	0.11	0.23	0.16	0.13	0.43	0.22	0.41	0.49	0.52	1	0.40	0.57	–0.12	–0.05
*P* value	.13	.37	.23	.20	.22	.01	.07	.16	<.001	.01	<.001	<.001	<.001	—	<.001	<.001	.16	.55
**Comprehension-T2**																		
*r*	0.15	–0.06	0.08	0.02	0.15	0.21	0.27	0.15	0.39	0.13	0.32	0.22	0.32	0.40	1	0.31	–0.05	0.05
*P* value	.09	.53	.36	.80	.10	.02	<.001	.10	<.001	.12	<.001	.01	<.001	<.001	—	<.001	.58	.60
**Emotional response-T2**																		
*r*	0.18	0.07	–0.10	–0.04	0.15	0.27	0.21	0.17	0.53	0.40	0.22	0.32	0.59	0.57	0.31	1	0.08	0.06
*P* value	.04	.45	.23	.67	.08	<.001	.02	.05	<.001	<.001	.01	<.001	<.001	<.001	<.001	—	.35	.49
**IGD** **symptoms** **-T1**																		
*r*	0.23	0.29	–0.17	–0.03	0.06	–0.09	–0.14	0.06	0.17	0.38	–0.30	–0.10	0.06	–0.12	–0.05	0.08	1	0.54
*P* value	.01	<.001	.02	.75	.36	.29	.10	.48	.05	<.001	<.001	.24	.06	.16	.58	.35	—	<.001
**IGD** **symptoms** **-T2**																		
*r*	0.16	0.27	–0.15	–0.02	0.05	–0.03	–0.02	0.10	0.25	0.45	–0.19	–0.01	0.16	–0.05	0.05	0.06	0.54	1
*P* value	.07	<.001	.08	.79	.57	.77	.86	.24	<.001	<.001	.05	.90	.06	.55	.60	.49	<.001	—

^a^Not applicable.

### Cross-Lagged Model of B-IPQ Dimensions and IGD Symptoms at T1 and T2

**Figure 1:**
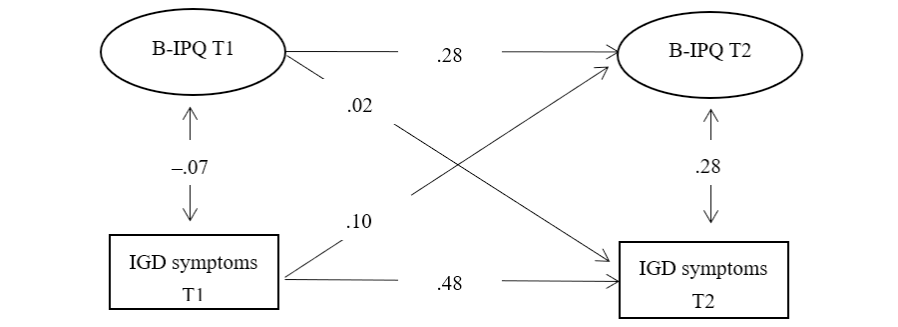
Cross-lagged model of Brief Illness Perception Questionnaire (B-IPQ) and internet gaming disorder (IGD) symptoms at T1 (baseline) and T2 (1-year follow-up) with standardized path coefficients.

The cross-lagged model fit the data well (χ^2^/*df*=2.28, CFI=.95, RMSEA=.06, SRMR=.05; Figure 1). IGD at T1 was positively associated with B-IPQ at T2 (B=.18, β=.10, 95% CI 0.01-0.19, *P*=.04). B-IPQ at T1 and IGD symptoms at T1 explained 8.8% of the variance in B-IPQ at T2. B-IPQ at T1 was not significantly associated with IGD at T2 (*P*=.42). B-IPQ at T1 and IGD symptoms at T1 explained 23.1% of the variance in IGD symptoms at T2. Standardized factor loadings of B-IPQ ranged from .34 to .81 at T1 and from .33 to .76 at T2 (Table 4).

**Table 4:** Factor loadings of Brief Illness Perception Questionnaire (B-IPQ) at T1 (baseline) and T2 (1-year follow-up).

Factor loadings	B	β	Composite reliability	*P* value
Consequence-T1 <--- B-IPQ T1	1.00	.38	-^a^	-^a^
Timeline-T1 <--- B-IPQ T1	1.22	.34	2.42	.01
Personal control-T1 <--- B-IPQ T1	1.21	.47	7.09	<.001
Treatment control-T1 <--- B-IPQ T1	1.71	.65	8.11	<.001
Identity-T1 <--- B-IPQ T1	1.83	.73	9.13	<.001
Concern-T1 <--- B-IPQ T1	2.17	.81	8.52	<.001
Comprehension-T1 <--- B-IPQ T1	1.45	.58	7.79	<.001
Emotional tesponse-T1 <--- B-IPQ T1	1.68	.68	8.19	<.001
Consequence-T2 <--- B-IPQ T2	1.00	.68	-^a^	-^a^
Timeline-T2 <--- B-IPQ T2	.61	.45	12.41	<.001
Personal control-T2 <--- B-IPQ T2	.43	.33	7.25	<.001
Treatment control -T2 <--- B-IPQ T2	.71	.48	10.19	<.001
Identity-T2 <--- B-IPQ T2	1.07	.74	14.13	<.001
Concern-T2 <--- B-IPQ T2	1.03	.73	14.43	<.001
Comprehension-T2 <--- B-IPQ T2	.65	.48	10.27	<.001
Emotional response-T2 <--- B-IPQ T2	.96	.76	14.80	<.001

^a^In the confirmation factor analysis, the loading of this factor was fixed at 1.

## Discussion

### Principal Findings

This study adopted a longitudinal design and conducted cross-lagged modeling analyses to demonstrate the relationship between illness representations of IGD and IGD symptoms. The results showed that only IGD symptoms at T1 predicted high levels of unfavorable illness representations at T2. Thus, hypothesis (2) was supported but not hypothesis (1). Indeed, people with greater IGD symptoms may be more likely to experience negative consequences of IGD in their daily life. For example, people with excessive and addictive use of internet games may suffer from mental and emotional disorders (eg, depression, anxiety, social anxiety, suicidal ideation), loneliness, interpersonal conflicts, and lack of offline social connection or support (eg, [18,19,21,24]). Since such people may have suffered from the IGD symptoms for at least 1 year, they have a better understanding of the fact that IGD can affect their daily life significantly and be chronic and cyclical. This is also consistent with the finding in the cross-sectional study in Macao [33]. In addition, people with IGD may have already experienced failure in attempting to regulate their internet gaming behaviors and thus perceive a great sense of helplessness and hopelessness as well as a low sense of self-control, self-efficacy, and personal control for their internet gaming behaviors and IGD symptoms. Such perceptions may partially explain why IGD may lead to some severe mental problems, such as depression and suicidal ideation (eg, [2,19]). However, illness representations did not show robust significant prediction for IGD symptoms in the model. According to the CSM, illness representations may directly affect risk perceptions and worry about IGD and self-regulation for IGD (eg, self-management, help-seeking), which may in turn influence one’s episode of IGD. Future work should test such potential outcomes and mechanisms.

Correlation analyses showed significant correlations between greater consequence, higher timeline, and low personal control at T1 and high levels of IGD symptoms at T2; in turn, IGD symptoms at T1 were also significantly correlated with these perceptions at T2, which seem to suggest longitudinal and interactive relationships between these perceptions and IGD. Other significant correlations included the correlations between negative emotional response at T1 and IGD symptoms at T2, between IGD symptoms at T1 and treatment control at T2, and between IGD symptoms at T1 and concern at T2. Similar correlations between these dimensions of illness representations and IGD symptoms were also found at the same time point. Not all the correlations were statistically significant. Since the dimensions represent different perceptions of a disease and some perceptions may be particularly relevant to the disease, it is reasonable that not all the dimensions of illness representations of IGD were significantly correlated with IGD symptoms. Consistently, the recent cross-sectional study also reported that only timeline cyclical, personal control, illness coherence, and emotional representations were significantly correlated with IGD symptoms [33]. Similar results were also found in previous studies of other diseases (eg, [32,40]). However, most of the correlations had small effect sizes. Thus, these relationships should be interpreted with caution. Since related studies are limited, conclusions regarding the relationships between different dimensions of illness representations and IGD symptoms cannot be drawn at this stage. More empirical studies are warranted.

It is interesting to find that the percentages of item agreement for each dimension of illness representations significantly increased at T2 compared with T1. A plausible explanation for this increase may be that both international and local societies have started to emphasize the adverse consequences of excessive use of the internet and video games in recent years. For example, the World Health Organization defined IGD as a mental disorder in 2018 [9]. In 2018, the Chinese government announced the establishment of a gaming regulator to limit the number of new online games, restrict playing time, and develop an age-restriction system [41]. Also, the local public media have repeatedly criticized video games for negatively influencing young people [41]. Thus, with the changes at the international, governmental, and social levels, local citizens, including college students, may have increasingly recognized the adverse consequences of overuse of internet games and known more about IGD.

The prevalence of IGD found in our study is consistent with another study with Chinese college students (10.3%) [15]. We further found that this prevalence did not significantly change from T1 to T2; a small number of non-IGD cases became IGD cases and vice versa. It may suggest stability of IGD status over time. It is different from another longitudinal survey with 283 Chinese university students that found a significant decrease in the prevalence (14.8% at T1 versus 9.9% at T2) [18]. The different results may be due to the differences in assessments, samples, and study period. However, since we had a relatively small and nonrepresentative sample, the findings should be interpreted with caution. Longitudinal and large-scale studies that monitor the prevalence and conversion rates of IGD across time are warranted.

The levels of illness representations of IGD changed significantly over time, while IGD did not change largely. It seems contradictory to our assumptions regarding their associations. One plausible explanation may be that although the protective factors of IGD (ie, unfavorable illness representations) increased over time, there may exist increasing independent risk factors (eg, daily life stress and academic stress) among the participants that could increase their risk of IGD or moderate the effect of illness representations on IGD. Future studies should investigate both risk and protective factors of IGD to better understand the change in IGD symptoms over time. These perceptions may have great effects on mental health and behavioral outcomes. For example, pessimistic views of IGD, such as low treatment control, may reduce ones’ help-seeking intention; a perception of low personal control may lead to anxiety and low self-efficacy. Future studies should test these mental health and behavioral outcomes.

### Strengths and Limitations

This study represents the first longitudinal study testing the relationships between illness representations and symptoms of IGD. It is also the first attempt at understanding illness representations of IGD among young people who are a group at high risk of IGD [13-17]. The findings suggest that IGD treatment should not only address IGD symptoms but also these perceptions, such as the perception of low personal control. Cognitive behavioral therapy and motivational enhancement therapy have been promising approaches to reduce addictive behaviors [42] and may help to enhance one’s motivation and skills to regulate their gaming behaviors.

Limitations of this study include the use of self-report measures, a nondiagnostic tool for IGD, and a small convenience sample. These might have affected the prevalence of IGD. The sample may differ from other populations considering some important demographic characteristics, such as age, educational level, majors, and regions. For example, the study only included medical students who might have extraordinary capabilities to assess their own health and possible threats of a disease. Thus, their perceptions of IGD may be different from other populations’ perceptions. Second, although the conduct of surveys within classroom settings helped to guarantee the survey’s quality, it might come with some limitations such as social pressure to participate. Third, only a 1-year follow-up was conducted. More waves and longer years of follow-up are warranted to better understand the stability of and changes in IGD and its consequences. Fourth, this study used the short version of IPQ. Although this version is more feasible in longitudinal surveys, future studies may use the long version of IPQ to validate our findings and test the reliability of each dimension of IPQ. Also, it was the first to use the B-IPQ in the context of IGD. Future studies should validate this scale well in the context of IGD. B-IPQ and IGD explained a relatively low amount of variance for each other. Future studies should include more potential important variables in the cross-lagged model. Fifth, it is worth noting that this study only investigated illness representations among gamers. Such perceptions may be different from those of nongamers [33]. In addition, it is a limitation that we did not know whether we included diagnosed cases. Since IGD is a newly defined disorder, diagnosis is rare, and treatment is still lacking in most local mental health services, there is little likelihood to include clinically diagnosed cases or those who know that they had IGD. Future studies may test whether illness representations have different implications between gamers and nongamers and between healthy individuals and those with diagnosed IGD. Finally, although the cross-lagged model is widely used to study causal influences in longitudinal panel data, some researchers have argued that, if stability of constructs is to some extent of a trait-like and time-invariant nature, the autoregressive relationships of the cross-lagged model may fail to adequately account for the causal influences [43]. As a consequence, the lagged parameters that are obtained with the cross-lagged model may not represent the actual within-person relationships over time, and this may lead to erroneous conclusions regarding the presence, predominance, and sign of causal influences. Other approaches should be used to validate the findings in future work.

### Conclusions

The findings suggest that illness representations of IGD increased over time and the level of IGD symptoms might affect ones’ illness representations of IGD. Based on the CSM, these illness representations may further influence gamers’ mental health, coping strategies, and behaviors related to the illness (eg, self-management, help-seeking). Future research may examine how illness representations of IGD would influence mental health and behavioral outcomes. Educational programs and psychological interventions are warranted to reduce the over-pessimistic perceptions about IGD based on the CSM structure and the self (eg, low personal control over IGD) among the gamers.

## Appendix

Multimedia Appendix 1 Measures.

## Abbreviations

B-IPQ: Brief Illness Perception Questionnaire
CFI: comparative fit index
CSM: common-sense model
DSM: Diagnostic and Statistical Manual of Mental Disorder
ICD: International Classification of Diseases
IGD: internet gaming disorder
RMSEA: root mean square error of approximation
SRMR: standardized root mean square residual
