# Supplementary material for: Relationship Between Illness Representations and Symptoms of Internet Gaming Disorder Among Young People: Cross-Lagged Model
Source: JMIR Serious Games. 2021 Nov 30;9(4):e28117. doi: 10.2196/28117 (PMC8672285; doi:10.2196/28117)
Supplement: Multimedia Appendix 1 [file games_v9i4e28117_app1.docx]

## Appendix A. Measures

**The Brief Illness Perception Questionnaire (B-IPQ)**

| How much does IGD affect the life of an IGD gamer? | | | | | | | | | | |
| --- | --- | --- | --- | --- | --- | --- | --- | --- | --- | --- |
| 0 | 1 | 2 | 3 | 4 | 5 | 6 | 7 | 8 | 9 | 10 |
| no effect at all |  |  |  |  |  |  |  |  |  | severely affects my life |
| How long do you think IGD symptoms of an IGD gamer will continue? | | | | | | | | | | |
| 0 | 1 | 2 | 3 | 4 | 5 | 6 | 7 | 8 | 9 | 10 |
| a very short time |  |  |  |  |  |  |  |  |  | forever |
| How much control do you feel one would have over IGD symptoms? | | | | | | | | | | |
| 0 | 1 | 2 | 3 | 4 | 5 | 6 | 7 | 8 | 9 | 10 |
| absolutely no control |  |  |  |  |  |  |  |  |  | extreme amount of control |
| How much do you think treatment can help IGD symptoms? | | | | | | | | | | |
| 0 | 1 | 2 | 3 | 4 | 5 | 6 | 7 | 8 | 9 | 10 |
| not at all |  |  |  |  |  |  |  |  |  | extremely helpful |
| How much do you think one gamer would experience symptoms from IGD? | | | | | | | | | | |
| 0 | 1 | 2 | 3 | 4 | 5 | 6 | 7 | 8 | 9 | 10 |
| no symptoms at all |  |  |  |  |  |  |  |  |  | many severe symptoms |
| How concerned are you about IGD symptoms? | | | | | | | | | | |
| 0 | 1 | 2 | 3 | 4 | 5 | 6 | 7 | 8 | 9 | 10 |
| not at all concerned |  |  |  |  |  |  |  |  |  | extremely concerned |
| How well do you feel you understand IGD symptoms? | | | | | | | | | | |
| 0 | 1 | 2 | 3 | 4 | 5 | 6 | 7 | 8 | 9 | 10 |
| don't understand at all |  |  |  |  |  |  |  |  |  | understand very clearly |
| How much does IGD affect you emotionally? (e.g. does it make you angry, scared, upset or depressed?) | | | | | | | | | | |
| 0 | 1 | 2 | 3 | 4 | 5 | 6 | 7 | 8 | 9 | 10 |
| not at all affected emotionally |  |  |  |  |  |  |  |  |  | extremely affected emotionally |

**IGD symptoms assessment by nine diagnostic criteria proposed in the DSM-5**

|  | No | Yes |
| --- | --- | --- |
| 1. Do you feel preoccupied with your gaming behavior? (Some examples: Do you think about previous gaming activity or anticipate the next gaming session? Do you think gaming has become the dominant activity in your daily life?) | 0 | 1 |
| 2. Do you feel more irritability, anxiety or even sadness when you try to either reduce or stop your gaming activity? | 0 | 1 |
| 3. Do you feel the need to spend increasing amount of time engaged gaming in order to achieve satisfaction or pleasure? | 0 | 1 |
| 4. Do you systematically fail when trying to control or cease your gaming activity? | 0 | 1 |
| 5. Have you lost interests in previous hobbies and other entertainment activities as a result of your engagement with the game? | 0 | 1 |
| 6. Have you continued your gaming activity despite knowing it was causing problems between you and other people? | 0 | 1 |
| 7. Have you deceived any of your family members, therapists or others because the amount of your gaming activity? | 0 | 1 |
| 8. Do you play in order to temporarily escape or relieve a negative mood (e.g., helplessness, guilt, anxiety)? | 0 | 1 |
| 9. Have you jeopardized or lost an important relationship, job or an educational or career opportunity because of your gaming activity? | 0 | 1 |
